# Daily temperature and mortality: a study of distributed lag non-linear effect and effect modification in Guangzhou

**DOI:** 10.1186/1476-069X-11-63

**Published:** 2012-09-14

**Authors:** Jun Yang, Chun-Quan Ou, Yan Ding, Ying-Xue Zhou, Ping-Yan Chen

**Affiliations:** 1 Department of Biostatistics, School of Public Health and Tropical Medicine, Southern Medical University, Guangzhou, 510515, China; 2 Faculty of Medicine, Southern Medical University, Guangzhou, 510515, China

**Keywords:** Mortality, Temperature, China, Distributed lag non-linear model

## Abstract

**Background:**

Although many studies have documented health effects of ambient temperature, little evidence is available in subtropical or tropical regions, and effect modifiers remain uncertain. We examined the effects of daily mean temperature on mortality and effect modification in the subtropical city of Guangzhou, China.

**Methods:**

A Poisson regression model combined with distributed lag non-linear model was applied to assess the non-linear and lag patterns of the association between daily mean temperature and mortality from 2003 to 2007 in Guangzhou. The case-only approach was used to determine whether the effect of temperature was modified by individual characteristics, including sex, age, educational attainment and occupation class.

**Results:**

Hot effect was immediate and limited to the first 5 days, with an overall increase of 15.46% (95% confidence interval: 10.05% to 20.87%) in mortality risk comparing the 99th and the 90th percentile temperature. Cold effect persisted for approximately 12 days, with a 20.39% (11.78% to 29.01%) increase in risk comparing the first and the 10th percentile temperature. The effects were especially remarkable for cardiovascular and respiratory mortality. The effects of both hot and cold temperatures were greater among the elderly. Females suffered more from hot-associated mortality than males. We also found significant effect modification by educational attainment and occupation class.

**Conclusions:**

There are significant mortality effects of hot and cold temperatures in Guangzhou. The elderly, females and subjects with low socioeconomic status have been identified as especially vulnerable to the effect of ambient temperatures.

## Background

The impact of weather on mortality has become public health significance, especially in light of climate change and rising frequency of adverse weather events (e.g., heat waves and floods). The relationship between temperature and mortality are extensively documented, with excess morality during days with extremely low or high ambient temperatures [1-6]. Substantive evidence has shown a delay between changes in daily temperature and changes in mortality, while the lag period considered are not consistent in different studies. Previous studies usually examined the effects for a single lag [4,7], and some studies chose *a priori* a lag period and assessed a cumulative effect [3,5]. To date, there is lack of a criterion for selecting the optimal lag. Exploring lag distribution of effects may provide some information for selecting an appropriate time frame when assessing temperature effects.

It has been shown that the association between temperature and mortality varied greatly by climate, geographic regions and populations [6,8,9], which indicates that it is necessary to assess the impacts of temperature in various regions. The relationship of temperature and mortality had been extensively studied in Europe [1,10,11] and the United States [3,4,6,12]. Only a limited number of studies were conducted in developing countries or tropical/subtropical regions [7,13-16]. More studies on health effects of weather in these regions are required to fully assess global impact of climate change and guide local public health policy.

To develop public health policies that protect those persons most vulnerable to extreme temperatures, researchers have identified factors that confer susceptibility. Previous studies indicated that black, the elderly and females were especially susceptible populations [8,17,18], while vulnerability by socioeconomic factors remains unclear. A few studies suggested that those with less education were at higher risk of temperature-related mortality [7,19,20], but some other studies showed little or no evidence for effect modification by area-based [21,22] or individual-based measures of education level [23,24]. Researchers have called for further research on the role of education level and other socio-economic measures to understand inequalities in health impact of ambient temperatures.

Guangzhou is the largest metropolis in Southern China with the latitude of 23° 7′ N. It is in a typical subtropical climate with mild winter and hot summer. In the present study, we sought to examine the association between daily ambient temperature and mortality in Guangzhou and identify the effect modification of temperature by individual characteristics, including age, sex, educational attainment and occupation class.

## Methods

### Health and environmental data

The Guangzhou Bureau of Health provided individual information for 112,280 deaths from 1 January 2003 to 31 December 2007, including date of birth, date of death, cause of death, sex, educational attainment and occupation. The underlying cause of death was classified by the Tenth Revision of the International Classification of Diseases (ICD-10). We considered non-accidental mortality (ICD-10: A00-R99), mortality due to cardiovascular diseases (I00-I99) and respiratory diseases (J00-J99), and three subcategories, including chronic obstructive pulmonary diseases (J40-J47), stroke (I60-I69) and ischemic heart diseases (I20-I25). Daily number of deaths was summarized by the underlying cause of death, sex, age group (0–64, 65–74, 75–84, 85 years old or above), educational attainment and occupation class, respectively. Educational attainment was defined as the highest degree of schooling completed before death. In the present study, it was classified into three groups: none (i.e. illiterate or semiliterate), primary education and secondary or higher education. Occupation was classified into unemployed (including housewife), blue-collar workers and white-collar workers.

We obtained the following meteorological data from China Meteorological Data Sharing Service System [25]: daily values of minimum, mean and maximum temperature, relative humidity and barometric pressure in Guangzhou. Weather data were collected from the only basic weather station in Guangzhou, Wushan Station. The Guangzhou Bureau of Environmental Protection provided air pollution data for 24-hour average concentrations of particulate matter with diameters less than 10 μm (PM_10_), nitrogen dioxide (NO_2_) and sulphur dioxide (SO_2_). These criteria pollutants have been associated with cardio-respiratory mortality [26]. The Ethics Committee of Southern Medical University where this study was conducted has approved the study proposal.

### Statistical methods

We fitted a distributed lag non-linear model (DLNM) to assess the association between daily number of deaths and ambient temperature. DLNM, proposed recently by Gasparrini et al. [27] is a flexible model to describe simultaneously a non-linear exposure-response relationship and delayed effect. It is appropriate to evaluate the characteristic of temperature-associated mortality in that temperature and mortality has shown a J-, W-, V- or U-shaped association [27-29]. A Poisson regression with quasi-Poisson function for daily counts of deaths was constructed, which was specified as

*Log*[*E*(*Y*_*t*_)] *= α + NS*(Time,5*7) *+ NS*(*Hum*_*t*_*,3*) *+ NS*(*Press*_*t*_*,3*) *+ NS*(*PM*_*10t*_*,3*) *+ NS*(*SO*_*2t*_*,3*) *+ NS*(*NO*_*2t*_*,3*) *+ γDow*_*t*_ *+ νHoliday*_*t*_ *+ βTemp*_*t*_*,*_*l*_

where *Y*_*t*_ is the observed daily deaths at day *t* (*t* = 1,2,3…1826); *α* is the intercept; *NS*(*.*) means a natural cubic spline; 7 degrees of freedom (df) per year for time and 3 df for relative humidity (*Hum*), barometric pressure (*Press*), *PM*_*10*_, *NO*_*2*_ and *SO*_*2*_ at the current day were recommended by several previous studies [13,16,27]. Dichotomous variables indicating day of the week (*Dow*) and public holidays (*Holiday*) are also included in the model. *Temp*_*t*__*l*_ is a matrix produced by DLNM to model non-linear and distributed lag effects of ambient temperature over the current day (lag 0) to lag *l* days, and *β* is vector of coefficients for *Temp*_*t,l*_; The maximum lag *l* was set to 25 days to explore the lag structure of temperature effect [17]. The median value of temperature was used as the reference value to calculate the relative risks. Akaike’s Information Criterion for quasi-Poisson (Q-AIC) was used to choose the df for temperature and lag [13,30]. The final composition of the function was a natural cubic spline of temperature with 5 df and a natural cubic spline with 5 df for lag days.

We fitted a model for each combination of ambient temperature measures (daily minimum, mean and maximum temperature) and mortality types using the above steps. The Q-AIC was used to choose the temperature measure that best predicted mortality. Mean temperature generally gave the lowest Q-AIC values based on our data Additional file (see 1), and mean temperature provides more easily interpreted results in a policy context and is more familiar to the public. Therefore, we chose mean temperature for subsequent analyses. We calculated percentage change in mortality risk comparing the first to the 10th percentile (cold effect) and the 99th to the 90th percentile (hot effect) of daily mean temperatures [17].

In order to identify subpopulations that are more susceptible to the effects of cold and hot temperatures, we conducted stratified analyses by sex, age group, educational attainment and occupation class. Furthermore, we used the case-only approach to determine the statistical significance of effect modification by testing the interaction between temperature and individual characteristics of interest. The case-only approach only examines cases. A logistic regression model was constructed with binary variable of individual characteristic of deaths as the dependent variable and mean temperature as the independent variable. The case-only approach has some apparent advantages over conventional time-series methods in assessing the interaction between a time-dependent variable and a time-fixed factor, such as practical simplification of modeling and reduction of potential time-invariant confounders related to death (e.g., smoking) [31,32]. In previous case-only studies, a dummy variable (e.g., an indicator of extreme temperature) or a linear term of temperature was included in the model [32,33]. However, the interaction between temperature and individual characteristics may be non-linear since the main effect of temperature on mortality is typically non-linear [4,8,27,34]. In the present study, we applied the distributed lag non-linear model to the case-only approach, which can be more flexible to determine whether individual characteristics modify the non-linear effect of temperature. The seasonal pattern of mortality was captured by including a sin and cosine term with a 365.24-day period in the model [32,33].

All statistical analyses and modeling were completed in R version 2.13.1 [35].

## Results

During the study period from January 1, 2003 to December 31, 2007, average daily maximum temperature was 27.6°C, mean temperature 23.0°C, minimum temperature 19.8°C in Guangzhou. The 1st, 10th, 90th and 99th percentile of daily mean temperatures was 8.2°C, 13.8°C, 29.9°C and 32.0°C, respectively. The mean concentrations of PM_10_, NO_2_ and SO_2_ were 88.2 μg/m^3^, 73.2 μg/m^3^ and 59.3 μg/m^3^, respectively. Among the total of 112,280 non-accidental deaths, 43,393 persons (38.7%) died from cardiovascular diseases and 21,071 (18.8%) from respiratory diseases. The descriptive statistics of daily number of deaths can be found in Table 1.

**Table 1:** Descriptive statistics of daily weather conditions and mortality from 2003 to 2007 in Guangzhou, China

**Variables**	**Minimum**	**Maximum**	**25th percentile**	**Median**	**75th percentile**	**Mean**	**Standard Deviation**
**Daily meteorological measures**							
Maximum temperature(°C)	7.2	39.1	23.4	28.8	32.6	27.6	6.2
Mean temperature(°C)	6.3	34.2	18.6	24.4	28.0	23.0	6.1
Minimum temperature(°C)	2.1	30.4	15.3	21.1	25.0	19.8	6.2
Relative humidity (%)	20.0	97.0	64.0	72.0	80.0	80.0	12.9
Atmospheric pressure(hPa)	988.7	1027.2	1003.4	1008.5	1014.0	1008.7	6.8
**Daily concentrations of pollutants**							
PM_10_(μg/m^3^)	7.0	370.1	52.1	80.0	114.6	88.2	48.5
NO_2_(μg/m^3^)	24.7	281.3	48.0	65.8	89.9	73.2	34.0
SO_2_(μg/m^3^)	6.1	237.3	29.3	49.7	80.3	59.3	39.6
**Daily number of deaths**							
Non-accidental mortality	32	233	52	59	69	62	13.6
Cardiovascular mortality	6	102	19	23	28	24	7.4
Respiratory mortality	2	46	8	11	14	12	4.5
All other mortality	8	85	22	26	30	26	6.1

**Figure 1:**
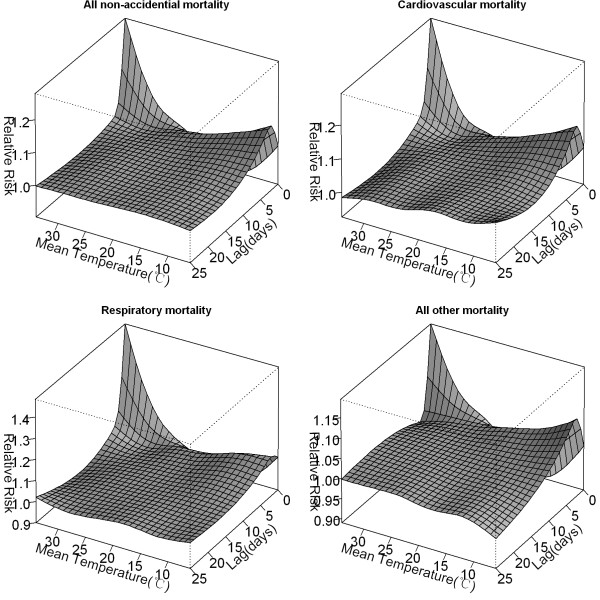
**Relative risks of mortality categories by daily mean temperature(°C) and days of lag.** The reference value was median temperature (24.4°C).

**Figure 2:**
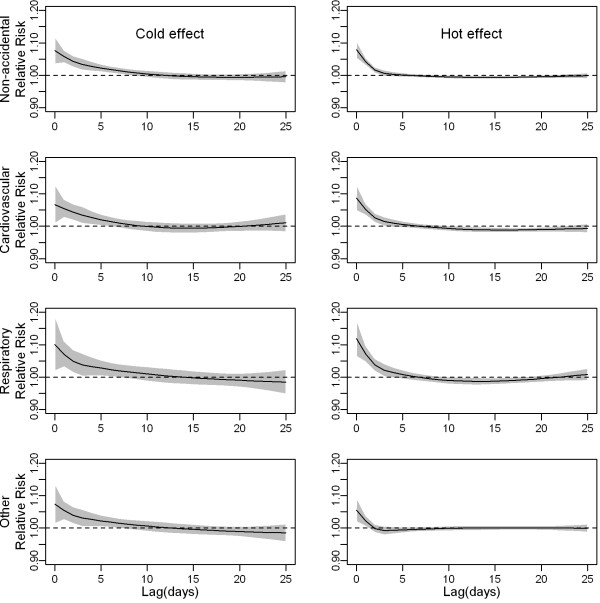
**The effect of mean temperature(°C) on mortality categories along days of lag.** The black lines are relative risks of mortality comparing the first to the 10th percentile (cold effect) and the 99th to the 90th percentile (hot effect) of temperatures, and grey regions are 95% confidence intervals.

The distributed non-linear lag surface revealed a non-linear relationship between temperature and mortality, with higher mortality risk at hot and cold temperatures (Figure 1). Figure 2 shows lag structures of temperature effect over the same day to 25 days previous. We observed immediate effects of cold and hot temperatures on mortality with the strongest and statistically significant effects on the current day (lag 0). Hot effects usually disappeared after approximately 4–5 days. Cold effects could persist for 10–12 days. The initial increase in mortality risk related to hot temperatures was followed by a decrease, consistent with short-term mortality displacement (i.e., harvesting), while no apparent harvesting was found for cold effects (Figure 2).

**Figure 3:**
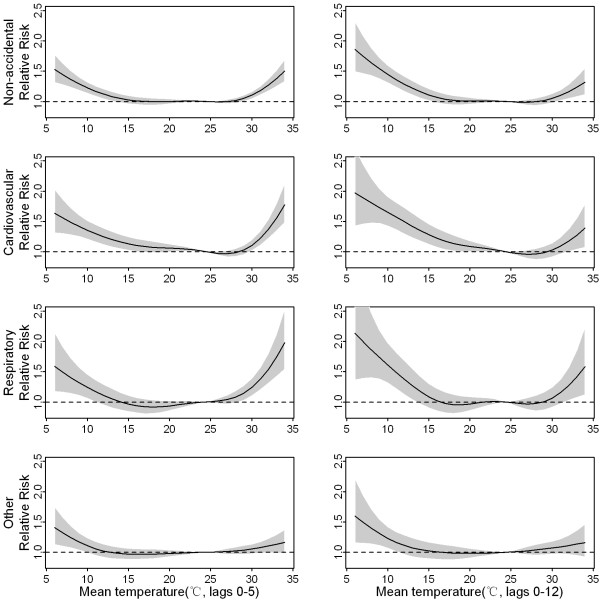
**The dose–response curve of daily mortality and mean temperature (°C) over lag 0–5 and lag 0–12 days.** The reference value was median temperature (24.4°C).

Based on the lag structures of hot and cold effects, we presented cumulative effects of hot temperatures at lag 0–5 days and cumulative cold effects at lag 0–12 days. The dose–response curve showed that both cold and hot effects seemed to be non-linear for some mortality categories (Figure 3). Therefore, we estimated relative effects of temperatures based on specific portions of the curve. Hot temperatures were associated with a 15.46% (95% confidence interval: 10.05% to 20.87%) increase in non-accidental mortality risk comparing the 99th to the 90th percentile of daily mean temperatures. Cold temperatures were associated with a 20.39% (11.78% to 29.01%) increase in non-accidental mortality comparing the first to the 10th percentile of temperatures. Stronger association was found with mortality due to cardiovascular and respiratory diseases, particularly with mortality due to ischemic heart diseases. Positive but non-significant effects of cold and hot temperatures were found for all other mortality (Table 2).

**Table 2:** Percentage increase (%) in mortality risk associated with hot and cold temperatures by causes

**Cause**	Hot effect*	Cold effect*
	**(95%CI)**	**(95%CI)**
**Non-accidental**	15.46(10.05 to 20.87)	20.39(11.78 to 29.01)
**Cardiovascular diseases**	20.89(12.24 to 29.54)	23.59(11.33 to 35.85)
Ischemic heart disease	30.01(12.98 to 47.04)	20.95(0.53 to 41.39)
Stroke	15.21(1.39 to 29.03)	11.77(−8.29 to 31.83)
**Respiratory diseases**	29.82(17.01 to 42.64)	34.82(16.89 to 52.75)
Chronic obstructive pulmonary diseases	21.38(5.56 to 37.20)	25.06(5.07 to 45.04)
**All other causes**	5.57(−1.43 to 12.57)	10.41(−5.02 to 25.82)

* The effects were presented by percentage increase (%) in mortality risk comparing the 99th to the 90th percentile (hot effect) and the first to the 10th percentile (cold effect) of temperatures.

To explore the potential confounding of air pollution, we performed a sensitivity analysis with adding one air pollution variable at a time or excluding all pollution variables from the model. There was only slight change in the estimate of temperature effect compared to the model controlling for three air pollution variables together Additional file (see 2), indicating that the confounding by air pollution was very small if there is any.

We evaluated the effects of temperatures on non-accidental mortality for subpopulations (Table 3) and further examined the statistical significance of effect modification by individual characteristics (Table 4). Hot effects were significantly larger for females than males. By contrary, males were at higher risk of cold effects compared to females but the difference was non-significant. The point estimates of temperature effects generally increased with age. There was statistically significant difference between the oldest age group (≥85 years old) and the youngest age group (<65 years old). A trend of decreased temperature-related mortality risk with increased education level was observed. No education conferred significant susceptibility. As to occupation class, blue-collar workers suffered more from hot and cold effects compared to white-collar worker, and the difference were statistically significant.

**Table 3:** Hot and cold effect by sex, age, educational attainment and occupation class

**Variable**	Hot effect*	Cold effect*
	**(95%CI)**	**(95%CI)**
Sex		
Male	8.05(1.82 to 14.28)	31.21(20.19 to 42.23)
Female	24.92(16.61 to 33.23)	16.53(4.79 to 28.27)
Age (years)		
0-64	2.04(−6.02 to 10.11)	8.49(−5.78 to 22.75)
65-74	10.40(0.68 to 20.12)	26.52(12.70 to 40.33)
75-84	13.75(4.83 to 22.67)	23.61(8.27 to 38.95)
85+	17.33(6.05 to 29.82)	28.62(10.71 to 46.53)
Educational attainment		
No education	25.03(11.81 to 38.25)	30.16(8.65 to 51.67)
Primary school	16.94(9.51 to 24.87)	15.91(2.11 to 29.71)
High school or above	5.40(−2.44 to 13.24)	13.80(5.55 to 22.21)
Occupation class		
White-Collar	11.59(−1.56 to 24.74)	16.27(−6.02 to 38.57)
Blue-Collar	18.34(6.65 to 31.31)	35.65(21.81 to 49.48)
Unemployed	17.25(9.31 to 25.78)	8.37(−9.61 to 26.32)

* The effects were presented by percentage increase (%) in mortality risk comparing the 99th to the 90th percentile (hot effect) and the first to the 10th percentile (cold effect) of temperatures.

**Table 4:** Modification by individual characteristics of temperature effects using the case-only approach

	**Factors**	**Hot effect modification**	**Cold effect modification**
		**OR(95%CI)**	**OR(95%CI)**
Sex	Male(Reference group)	_	_
	Female	1.192(1.134 to 1.253)	0.951(0.838 to 1.064))
Age	0-64 years(Reference group)	_	_
	65-74 years	1.083(1.006 to 1.161)	1.075(0.891 to 1.259)
	75-84 years	1.103(1.008 to 1.198)	1.069(0.857 to 1.319)
	85 years or above	1.195(1.099 to 1.298)	1.187(1.068 to 1.306)
Education	None (reference group)	_	_
	Primary education	0.933(0.867 to 1.005)	0.903(0.757 to 1.049)
	Secondary or higher education	0.862(0.797 to 0.931)	0.849(0.702 to 0.996)
Occupation	White collar (reference group)	_	_
	Blue collar workers	1.095(1.027 to 1.163)	1.104(1.043 to1.166)
	Unemployed	1.080 (0.912 to 1.246)	0.953(0.748 to 1.158)

Odds ratio (OR) larger than 1 indicates positive effect modification, that is, the increased risk of mortality associated with hot or cold temperatures was larger for persons who had this characteristic compared to the reference group.

## Discussion

To quantify mortality effects of ambient temperature, linear threshold regression models were often used previously, which assumed linear or log-linear increase below and above the threshold. In fact, the dose–response curve of temperature and mortality is hardly linear below or above the threshold, so it is inappropriate to use linear threshold models to estimate the effects directly. Recently Gasparrini and Armstrong first rigorously developed DLNMs [27,28]. With unifying many of the previous methods in one unique framework, DLNMs are flexible enough to describe non-linear dependencies and delayed effects of exposure at the same time. Gasparrini et al. [27] speculated in their article that the DLNMs could be easily translated in other study design and regression models. In the present study, we fitted DLMNs to fully understand the dose–response function and lag effects of temperature. Furthermore, we applied DLNMs to the case-only approach. This allows sophisticated non-linear and delayed effect modification to be estimated and tested statistically.

We found significant impacts of hot and cold temperatures on mortality in Guangzhou, China. Hot temperatures had an acute but short-term effect, whereas the effect of cold temperatures lasted 10–12 days. Similar lag structures with characteristics of short term for hot effects and long duration for cold effects were observed in England [28], US [17,27] and Canada [29]. This finding suggests that a longer timeframe are required to capture the cold impact, and that it may be inappropriate to specify *a priori* identical timeframe for cold and hot exposure. Guo et al. [13] stated that use of short lags may underestimate cold effects, but it may overestimate hot effects. In the present study, we estimated the effects of hot and cold temperatures for two different lag periods.

Some evidence in the literature shows that the magnitude of temperature effects varied by climate and population. Notably, we found a significant cold effect with a 20.39% increase in mortality risk comparing 8.2°C to 13.8°C. An analysis in the subtropical region of Chiang Mai, Thailand reported a similar cold effect with a 19% increase of mortality risk over lag 0–13 days comparing 19.35°C to 24.7°C [14]. Multi-city studies reported a higher risk of mortality associated with cold exposure in regions having milder winter climates [4,34]. Thus, the public perception that hot regions do not suffer from cold weather is completely mistaken. Our findings highlight a need to strengthen the awareness of combating cold exposure in the public.

By analyzing in subgroups, many studies have found that the elderly were at higher risk of mortality associated with cold [4,10] or hot temperatures [4,10,19]. It may be due to their poorer physiological adaptation to changes in ambient temperatures. Our findings confirmed vulnerability of the elderly. An interesting finding is that females were more susceptible to hot but not cold temperatures compared to males, which is in agreement with the results in US [33], Europe [18,36] and Korea [37]. Gender difference may be dependent on location and population. For example, the impact of hot temperature on mortality was higher for women in Mexico, but higher for men in Sao Paulo [7].

Greater effects of ambient temperatures were observed for those with less education in the United States [19], Latin American [7], Korea [20,37] as well as in Shanghai, China [38]. However, no effect of education level on temperature-related mortality risk was found in California [23] and Australia [24]. Moreover, all previous findings were based on stratified analysis and it remains uncertain whether effect modification by education was statistically significant or not. In the present study, educational attainment was classified into three groups. Subjects with no education were significantly more susceptible to the effects of both hot and cold temperatures compared to other education groups. Additionally, to our knowledge, this is the first study to examine the potential effect modification by occupation class. We found that blue-collar workers were at significantly higher risk of temperature-related mortality than white-collar workers.

When analyzing by cause of death, effect estimates were markedly higher for cardiovascular and respiratory deaths compared to all non-accidental deaths, consistent with previous studies [5,13,17]. From a public health point of views, this finding is important since cardio-respiratory diseases are the leading cause of death in Guangzhou, accounting for 58% of all registered deaths during the study period. This increase in mortality is likely related to the failure in thermoregulation and the physiological changes in circulatory system [39].

Some limitations should be mentioned. Firstly, the data are only from one city, so it should be cautious to generalize the findings to other geographic areas and other climates. Secondly, we considered several major mortality categories but not very fine categories. Notably, as in all time-series studies on temperature effects, we assigned each individual the same exposure level measured by ambient daily mean temperature, which would bring about measurement errors because indoor temperature may be not closely correlated with outdoor temperature due to the use of air condition. Lastly, temperature-associated mortality was calculated by comparing the first to the 10th percentile and the 99th to the 90th percentile temperatures. This accounted for the effects of extreme temperatures in some way. However, further research aimed at the definition of warning systems and prevention programs should assess the potential added effect of heat waves and cold spells especially in terms of persistency of extreme conditions, frequency of events and adaptation of the population throughout the summer season.

## Conclusions

Our results confirmed that cold and hot temperatures were associated with increased risk of mortality in the subtropical city of Guangzhou, China. The elderly, females and subjects with low socioeconomic status have been identified as especially susceptible to temperature-associated mortality. The findings can contribute to focus community and individual prevention programs targeting on mitigating weather-related mortality.

## Abbreviations

CI: Confidence interval; DLNMs: Distributed lag non-linear models; ICD 10: International classification of diseases tenth revision; NO_2_: Nitrogen dioxide; OR: Odds ratio; PM_10_: Particulate matter with aerodynamic diameters less than 10 μm; Q-AIC: Quasi-likelihood Akaike information criteria; SO_2_: Sulphur dioxide.

## Competing interests

The authors declare that they have no competing interests.

## Authors’ contributions

CQ and PY initiated the study and collected the data. JY, YD and YX cleaned the data and performed statistical analysis. JY and CQ drafted the manuscript. All authors read and approved the final manuscript.

## Supplementary Material

Additional file 1 Quasi-likelihood Akaike information criteria (Q-AIC) values for the relationship between temperature measures and mortality categories. Click here for file

Additional file 2 The cumulative effect of cold and hot temperatures, with and without pollution adjustment. Click here for file
