# Supplementary material for: Daily temperature and mortality: a study of distributed lag non-linear effect and effect modification in Guangzhou
Source: Environ Health. 2012 Sep 14;11:63. doi: 10.1186/1476-069X-11-63 (PMC3511876; doi:10.1186/1476-069X-11-63)
Supplement: Additional file 1 — Quasi-likelihood Akaike information criteria (Q-AIC) values for the relationship between temperature measures and mortality categories. [file 1476-069X-11-63-S1.doc]

**Additional file 1 Quasi-****likelihood Akaike information criteria (Q-AIC) values for the relationship between temperature measures and mortality categories**

| **Temperature measure** | **Mortality categories** | | | |
| --- | --- | --- | --- | --- |
|  | **Non-accidental** | **Cardiovascular** | **Respiratory** | **All other mortality** |
| Minimum temperature | 12598 | 10585 | 9194 | 10758 |
| Maximum temperature | 12557 | 10565 | 9167 | 10760 |
| Mean temperature | 12517 | 10549 | 9152 | 10742 |
