# Supplementary material for: Daily temperature and mortality: a study of distributed lag non-linear effect and effect modification in Guangzhou
Source: Environ Health. 2012 Sep 14;11:63. doi: 10.1186/1476-069X-11-63 (PMC3511876; doi:10.1186/1476-069X-11-63)
Supplement: Additional file 2 — The cumulative effect of cold and hot temperatures, with and without pollution adjustment. [file 1476-069X-11-63-S2.doc]

**Additional file 2 The cumulative effect of cold and hot temperatures, with and without pollution adjustment**

| **Pollution adjustment** | **Hot effect**  **(95%CI)** |  | **Cold effect**  **(95%CI)** |
| --- | --- | --- | --- |
| None | 16.51(11.19 to 21.83) |  | 19.31(11.31 to 27.30) |
| Adjusted by PM_10_ | 15.48(10.10 to 20.86) |  | 20.19(11.68 to 28.71) |
| Adjusted by NO_2_ | 15.63(10.27 to 20.99) |  | 19.82(11.34 to 28.30) |
| Adjusted by SO_2_ | 15.89(10.52 to 21.26) |  | 20.25(11.73 to 28.76) |
| Adjusted by PM_10_ ,NO_2_ and SO_2_ | 15.46(10.05 to 20.87) |  | 20.39(11.78 to 29.01) |

PM_10_: particulate matter with aerodynamic diameters less than 10 μm; NO_2_: nitrogen dioxide; SO_2_: sulphur dioxide
